# The computational analysis of tumor cell sensitivity to supertarget deletion

**DOI:** 10.18699/vjgb-26-02

**Published:** 2026-03

**Authors:** D.A. Chetverina, N.Y. Kozelchuk, D.V. Lomaev, A.A. Shtil, М.M. Erokhin

**Affiliations:** Institute of Gene Biology Russian Academy of Sciences, Moscow, RussiaInstitute of Gene Biology Russian Academy of Sciences, Moscow, Russia; Institute of Gene Biology Russian Academy of Sciences, Moscow, Russia; Institute of Gene Biology Russian Academy of Sciences, Moscow, RussiaInstitute of Gene Biology Russian Academy of Sciences, Moscow, Russia; N.N. Blokhin National Medical Research Center of Oncology, Moscow, Russia; Institute of Gene Biology Russian Academy of Sciences, Moscow, Russia

**Keywords:** tumors, cancer, oncomarkers, Dependency Map, DepMap, transcription, mutations, SMARCA2, SMARCA4, APP, FOXA1, ATP6V0A2, ATP6V0A1, bioinformatics, database analysis, personalized medicine, опухоли, рак, онкомаркеры, Dependency Map, DepMap, транскрипция, мутации, SMARCA2, SMARCA4, APP, FOXA1, ATP6V0A2, ATP6V0A1, биоинформатика, анализ баз данных, персонализированная медицина

## Abstract

Gene mutations and altered epigenetic regulation of gene expression are characteristic features of malignant neoplasms. Combinations of these abnormalities form molecular features of individual tumors. In the large-scale Dependency Map (DepMap) project, the broad panels of human tumor cell lines are being tested for sensitivity to single gene inactivation. Using DepMap data, we have previously identified a set of genes termed supertargets, the deletion of which significantly reduced the survival of cells of a particular tissue origin while minimally impairing the unrelated cell lines. In the present study, we determined the factors of viability (inhibition of proliferation or death) of cell lines in which the supertarget genes have been deleted. We found that, in 79 % of cases, the reduced survival may be caused by epigenetic changes of gene expression. In the remaining 21 % of cases, it is associated with altered gene structure. Three groups containing different types of gene expression alterations can be distinguished. In the first group, the reduced cell survival correlated with a higher expression of the supertarget gene (e. g., SOX10 and HNF1B). In the second group, a gene different from the deleted supertarget was overexpressed (gene pairs: FOXA1 and SPDEF, TP63 and SERPINB13, etc.). The third group was characterized by correlations between low expression of a certain gene and tumor cell sensitivity (e. g., FAM126A and FAM126B, SMARCA2 and SMARCA4). The genetic changes included GOF mutations (KRAS, BRAF genes, etc.), LOF mutations (STAG1, SMARCA2 genes, etc.), gene fusions (BCR-ABL1, PAX3-FOXO1, etc.), and amplification (CPM, BEST3, etc.). Therefore, many different molecular mechanisms act as predictors of tumor cell response to inhibition of supertarget genes.

## Abstract (ru)

Мутации генов и изменения эпигенетической регуляции экспрессии генов являются характерными признаками злокачественных новообразований. Сочетания данных нарушений формируют биологические особенности индивидуальных опухолей на молекулярном уровне. Разработка стратегий персонализированного лечения требует глубокого понимания молекулярных «портретов» отдельных опухолей. В рамках крупномасштабного проекта Dependency Map (DepMap) обширные панели линий опухолевых клеток человека тестируются на чувствительность к инактивации отдельных генов. Ранее на основе данных DepMap нами охарактеризованы гены, получившие название «супермишени», при делеции которых существенно снижена жизнеспособность клеток конкретного тканевого происхождения при минимальном нарушении жизнеспособности других линий. В настоящем исследовании определены факторы низкой жизнеспособности (ингибирования пролиферации или гибели) клеточных линий при инактивации генов-супермишеней. В результате установлено, что в 79 % случаев низкая жизнеспособность может быть вызвана эпигенетическими изменениями в экспрессии генов. В остальных случаях (21 %) она вызвана нарушениями структуры генов. Исходя из полученных данных, можно выделить три группы, содержащие разного типа нарушения экспрессии генов. В первой группе низкая жизнеспособность клеток коррелирует с повышением экспрессии гена-супермишени (например, SOX10 и HNF1B). Во второй группе детектируется гиперэкспрессия гена, отличного от делетируемой супермишени (пары генов FOXA1 и SPDEF, TP63 и SERPINB13 и др.). Третья группа характеризуется корреляциями между пониженной экспрессией определенных генов и чувствительностью опухолевых клеток (пары генов FAM126A и FAM126B, SMARCA2 и SMARCA4 и др.). Наблюдаемые генетические изменения включали GOF-мутации (KRAS, BRAF и др.), LOF-мутации (STAG1, SMARCA2 и др.), слияние генов (BCR-ABL1, PAX3-FOXO1 и др.) и амплификации (CPM, BEST3 и др.). Таким образом, разные молекулярные механизмы выступают предикторами ответа опухолевых клеток на ингибирование генов-супермишеней

## Introduction

The current approach to antitumor therapy involves identification of molecular mechanisms specific to a particular tumor type. The principle of targeted therapy is the inactivation of these factors to achieve an antiproliferative effect and/or cell death with minimal damage to non-malignant counterparts (Verma, 2012; Pfohl et al., 2021). The search for tumor-specific targets includes the screening of cell lines of various tissue origin on broad panels. The most large-scale project is the Dependency Map (DepMap, https://depmap.org/), which analyzes inactivation of an individual gene via RNAi and CRISPR/ Cas9 technologies (Tsherniak et al., 2017; Arafeh et al., 2025).

Previously, using the DepMap resource, we studied 27 histological types of tumors; for each type, five genes were identified, the CRISPR/Cas9-mediated knockout of which reduced the viability (i. e. inhibition of proliferation or death) of cells of a particular type (Chetverina et al., 2023). These genes, termed “supertargets”, can be considered promising candidates for personalized therapy

In this study, we used the DepMap resource to dissect the genetic and epigenetic changes that correlate with reduced cell viability upon supertarget deletion. The identified factors can be used to predict the sensitivity of cells of an individual tumor type to inactivation of a particular target.

## Materials and methods

Analysis of tumor cell viability was performed using the DepMap database (https://depmap.org/portal/, Tsherniak et al., 2017), Public 22Q4+Score, Chronos release (https://doi. org/10.25452/figshare.plus.27993248.v1). Viability was analyzed using the “Gene effect” value. To attribute low viability to a factor, two criteria were used: the value of the significance parameter (Importance) >10 %, and the degree of reliability (Student’s t-test) <0.01. Gene expression levels were analyzed using DepMap Expression Public Release 22Q4 (https:// depmap.org/portal/data_page/?tab=allData&releasename= DepMap%20Public%2024Q4&filename=OmicsExpress ionProteinCodingGenesTPMLogp1.csv) The gene copy number was determined using the DepMap Copy Number Public version 22Q4 (https://depmap.org/portal/data_ page/?tab=allData&releasename=DepMap%20Public%20 24Q4&filename=OmicsCNGene.csv).

The STRING database (https://string-db.org/, version 12.0) was used to analyze protein-protein interactions. Only interactions confirmed by biochemical methods were taken into account

## Results and discussion

**Low viability of tumor cell lines upon deletion of genes encoding supertargets**

Previously, we analyzed 27 histological tumor types to identify the supertarget genes, that is, the ones the knockout of which most specifically affected the viability of a particular tumor compared with other cancers. For each tumor type, the top five genes critical for survival were identified, yielding a total of 124 unique supertargets (nine genes were repeated twice in different tumors and one was found thrice) (Chetverina et al., 2023). To identify genetic and/or epigenetic changes that correlate with low cell viability upon knockout of an individual supertarget, we used the Importance parameter set by the Dep- Map project. The search for correlation factors included data on all cell lines in the database without grouping. The RNAseq data and genetic alterations in intact cell lines were compared with gene effect in cell lines with inactivated supertarget genes. The search was performed for each supertarget individually

A total of 167 associated correlations were found to link low viability of tumor cells (i. e. inhibited proliferation and/or death) with the knockout of supertarget genes. For a number of genes, two or more predictors of low viability were discovered whereas no reliable correlations were detectable for 23 out of 124 supertargets. Among the factors of low viability, the majority (132 cases) were associated with changes in gene expression, and 35 cases with altered gene structure.

**Gene expression abnormalities that correlate with low cell viability upon knockout of supertarget genes**

Overexpression of the supertarget. Detailed analysis revealed three groups of gene expression abnormalities that correlate with low cell viability upon supertarget knockout. In the first group (50 cases), low cell viability correlated with high expression of the supertarget (Table 1).

**Table 1.**
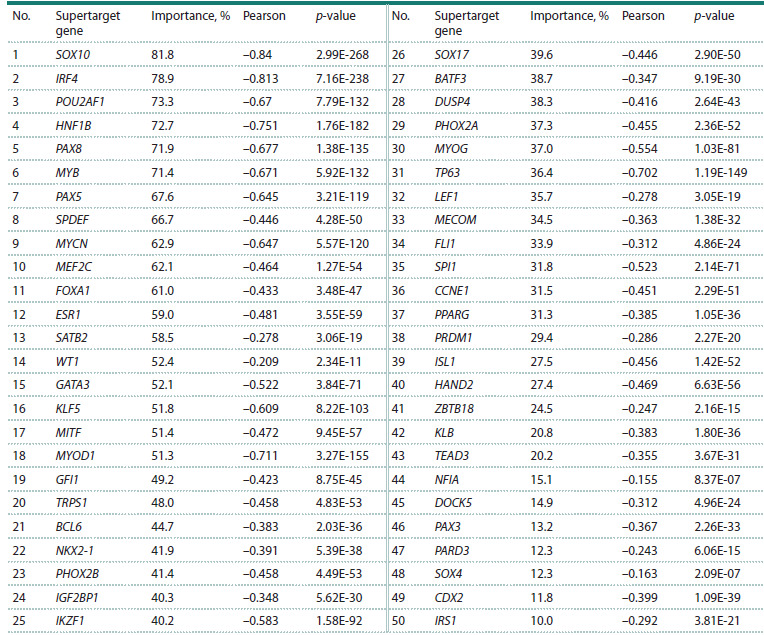
Low cell viability upon supertarget knockout correlating with a high level of expression of the same supertarget

**Fig. 1.**
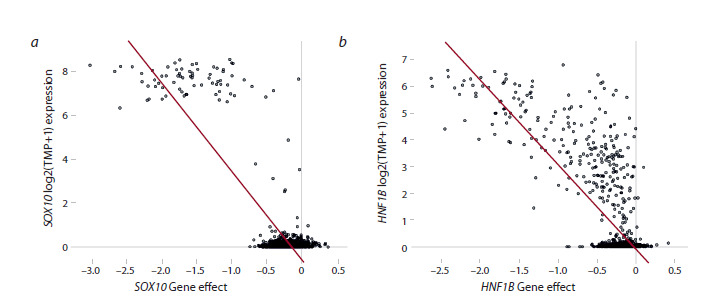
Overexpression of the supertarget gene and low viability of tumor cells Correlations between gene expression and low cell viability upon knockout of the SOX10 (a) and HNF1B (b) genes. The Y axis shows the expression level of the corresponding gene (log2(TPM+1)), the X axis shows the estimated Gene effect value reflecting cell viability upon knockout of the supertarget gene. The smaller the Gene effect value, the lower the viability and the higher the inhibition of proliferation and/or cell death of a given cell line upon inactivation of the corresponding gene. Here and in Tables 2–4: black dots show values for individual cell lines. The diagonal line is the regression.

Figure 1 shows two typical examples. The SOX10 gene (SRY-box transcription factor 10, Fig. 1a) has been previously identified as a supertarget in melanoma-derived cell lines. It encodes a transcription factor important for cell differentiation in the central and peripheral nervous system, melanocytes, and chondrocytes. Dysregulation of SOX10 is known to be associated with carcinogenesis (Bahmad et al., 2023).

The HNF1B gene (HNF1 homeobox B, Fig. 1b) has been identified as a supertarget for renal cancer cell lines. This gene encodes a homeobox-containing transcription factor important for the liver, kidney, and pancreatic development during embryogenesis (Chandra et al., 2021). Dysfunctions of HNF1B, including germline and somatic mutations, have been identified in a variety of solid tumors (Bártů et al., 2018).

Overexpression of a gene different from the inactivated supertarget. The second group of correlations contains 71 cases in which low cell viability upon supertarget deletion correlates with increased expression of a gene unrelated to the knockedout supertarget (Table 2).

**Table 2.**
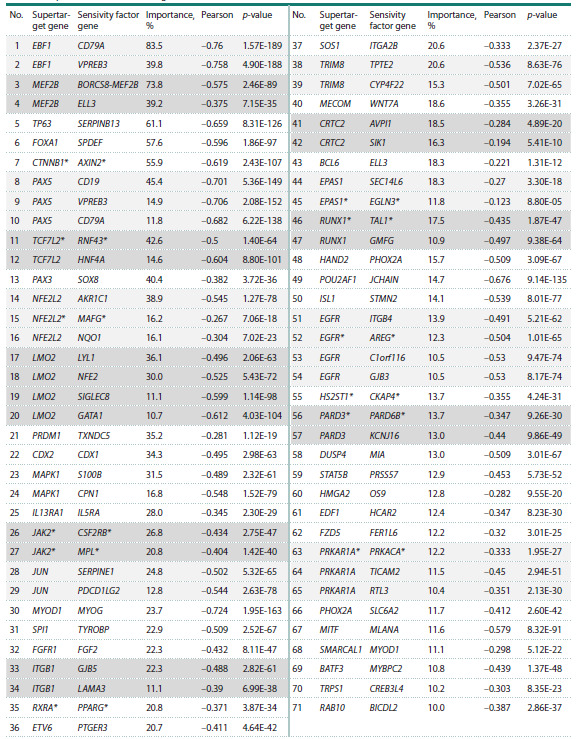
Correlation of low cell viability upon supertarget knockout with overexpression of an unrelated gene Note. The “*” symbol marks pairs of proteins for which direct interactions have been shown by biochemical methods (STRING database). Groups corresponding to the same gene are shown in grey.

**Fig. 2.**
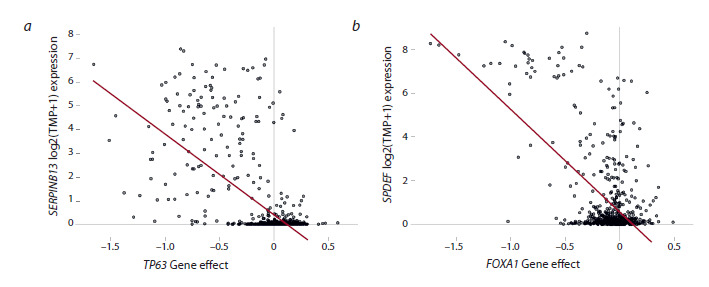
Cell viability correlates with expression of genes unrelated to the inactivated supertarget a – correlation between SERPINB13 expression and low cell viability upon TP63 deletion; b – correlation between SPDEF expression and low cell viability upon FOXA1 deletion.

In 65 cases, correlations were found between low cell viability upon knockout of a supertarget gene and elevated expression of an unrelated gene. An example of such dependence is the TP63 and SERPINB13 pair (Fig. 2a). The TP63 gene, which we assigned as a supertarget for upper aerodigestive cancer cell lines (Chetverina et al., 2023), encodes the p63 protein, a transcriptional regulator, like its homologs p53 and p73, involved in numerous processes in tumor biology (Sadu Murari et al., 2025). The SERPINB13 gene encodes a serine protease antagonist that inhibits the activity of cathepsins K and L (Jayakumar et al., 2003; Welss et al., 2003). Recent studies have shown that SERPINB13 may act as an oncogene in squamous cell lung cancer (Zhang et al., 2025). However, to our knowledge, there are no data on functional interactions between p63 and SERPINB13.

In six cases, we observed a correlation between low cell viability upon supertarget knockout and overexpression of a gene classified as a supertarget in the previous screening: the FOXA1–SPDEF, MYOD1–MYOG, TCF7L2–HNF4A, RXRA– PPARG, HAND2–PHOX2A, SMARCAL1–MYOD1 gene pairs. For example, the FOXA1 (forkhead box A1) and SPDEF (SAM pointed domain containing the ETS transcription factor, Fig. 2b) genes have been identified as supertargets in breast cancer cell lines (Chetverina et al., 2023). The FOXA1 protein activates the SPDEF gene, while SPDEF activates FOXA1 transcription (Buchwalter et al., 2013; Paranjapye et al., 2020). Although there are no data on direct interactions between FOXA1 and SPDEF proteins, their genes are located in the same regulatory cluster, which also includes the genes for estrogen receptor (ER) and GATA3 transcription factor, both important in mammary gland carcinogenesis (Lemieux et al., 2017).

The detected dependencies may be due to physical and functional interactions between proteins encoded by identified gene pairs. To address this possibility, we tested the presence of physical contacts between the proteins encoded by the identified genes using the STRING database. We found that 12 gene pairs encoded direct protein–protein interactors (Table 2). It can be assumed that, for other gene pairs, there could be an indirect effect, or else physical interactions between proteins have not yet been established

Low expression of a non-supertarget gene. The third group of correlations contained 11 cases in which low cell viability upon supertarget knockout correlated with low expression of a non-supertarget gene. Most frequently (nine cases), the low-expressed non-supertarget gene was a close homologue of the supertarget (Table 3).

**Table 3.**
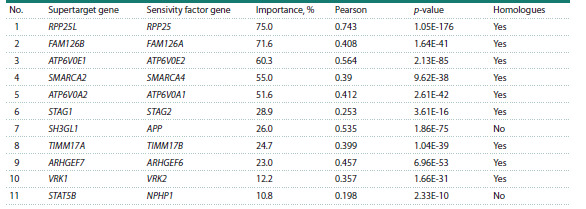
Low viability upon supertarget knockout correlates with low expression of an unrelated gene

**Fig. 3.**
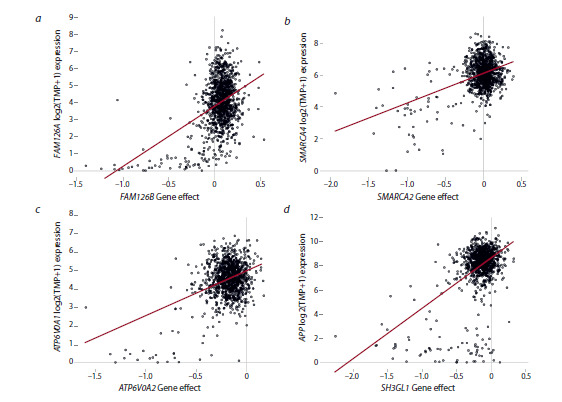
Low expression of a non-supertarget gene correlates with poor viability upon supertarget knockout a – correlation of FAM126A expression and cell sensitivity to FAM126B knockout; b – correlation of SMARCA4 expression and low viability upon SMARCA2 knockout; c – correlation of ATPV0A1 expression and sensitivity to ATPV0A2 knockout; d – correlation of APP expression and low cell sensitivity to SH3GL1 deletion.

Typical examples are shown in Figure 3a, b. Low cell viability upon FAM126B knockout correlated with the low expression of its homologue gene FAM126A (Fig. 3a). The FAM126A and FAM126B genes (also known as HYCC1 and HYCC2, stands for hyccin PI4KA lipid kinase complex subunit) encode the members of the PI4KIIIα/PI4KA protein kinase complex which regulates lipid composition and asymmetry of the plasma membrane (Suresh et al., 2024). Figure 3b demonstrates the correlation of low gene effect upon SMARCA2 (SWI/SNF related, matrix associated, actin dependent regulator of chromatin, subfamily A, member 2) knockout with low expression of its homologue SMARCA4. Proteins encoded by SMARCA2 and SMARCA4 are the components of the SWI/SNF chromatin remodeling complex. Altered structure and function of this complex have been often found in tumors (Nguyen et al., 2023; Reddy et al., 2023).

In the case of the ATP6V0A2 (ATPase H+ transporting V0 subunit a2) gene knockout, low cell viability correlated with low expression of its homologue ATP6V0A1 (Fig. 3c). The ATP6V0A2 and ATP6V0A1 genes encode a component of the V-ATPase proton channel, which maintains an electrochemical proton gradient across the plasma membrane. In addition to its main function, V-ATPase is involved in the Notch/Wnt pathway (Eaton et al., 2021). Detailed functions of ATP6V0A1/2 remain to be established.

**Fig. 4.**
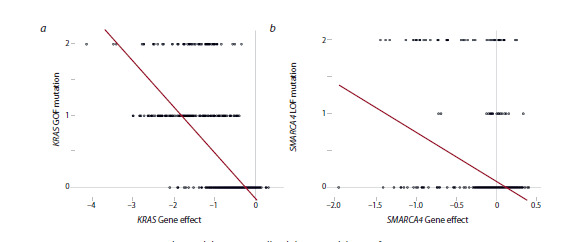
Genetic mutations correlate with low tumor cell viability upon deletion of supertarget genes a – correlation between the number of the KRAS mutations and cell sensitivity to KRAS knockout; b – correlation between the number of SMARCA4 mutations and cell sensitivity to SMARCA2 knockout

For SH3GL1 (SH3 domain containing GRB2 like 1) knockout, low viability correlated with low expression of the APP (amyloid precursor protein) gene (Fig. 4d). The SH3GL1 gene encodes endophilin A2 important for the dynamics of intracellular membranes, in particular, for endocytosis (Yang et al., 2023). The role of APP has been investigated in Alzheimer’s disease: an APP fragment (β-amyloid) forms characteristic plaques in the brain (Chen et al., 2024). There are indications of the involvement of APP in carcinogenesis, but its role has not been established conclusively (Lee et al., 2021). No interactions between SH3GL1 and APP have been reported, nor was their mutual role in tumor biology studied. Thus, our analysis allows to predict tumor cell response to deletion of a particular gene as well as to reveal previously unknown functional interactions between the gene products.Gene structure alterations. In 35 cases, correlations were observed between low cell viability upon supertarget knockout and altered gene structure. The types of abnormalities included point mutations (12 cases), gene fusions (13), and amplifications (10 cases) (Table 4).

**Table 4.**
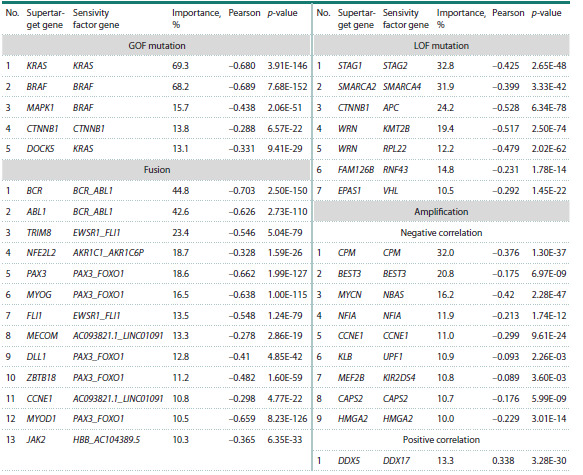
Changes in the structure of supertarget and other genes that correlate with low cell viability upon deletion of the supertarget

Figure 4a shows the correlation for the KRAS (Kirsten rat sarcoma virus) gene, which encodes a small GTPase, a key component of the signaling pathway activated by the interaction of epidermal growth factor with its receptor (Seres et al., 2025). KRAS-activating mutations (gain-on-function, GOF) correlated with the sensitivity of cells to KRAS knockout

The example in Figure 4b demonstrates the correlation between the low gene effect upon SMARCA2 knockout and the number of inactivating (loss-of-function, LOF) mutations in its close homologue SMARCA4. As indicated above, SMARCA2 and SMARCA4 are the components of the SWI/SNF chromatinremodeling complex. Increased cell sensitivity to SMARCA2 knockout correlated with LOF mutations in the SMARCA4 gene (Fig. 4b) and with low expression of SMARCA4 (Fig. 3b).

## Conclusion

A detailed analysis of sensitivity of human tumor cell lines to knockout of supertarget genes was performed. We use the term “supertarget” for genes, the inactivation of which, according to the DepMap database, significantly reduces the viability of tumor cells of a particular tissue origin. Most frequently, low cell viability correlated with the expression of particular genes, i. e. supertargets as well as unrelated genes. Also, cell viability can be affected by genetic mutations such as GOF, LOF, gene fusion and amplification. Data on functional interdependences can be used to test the sensitivity of tumor cells of different origin to inactivation of supertarget genes by conventional and investigational drugs.

The established correlations are relevant to the development of personalized treatment strategies based on biological characteristics of the patient’s tumor, that is, its molecular “portrait”. Interpretation of tumor sensitivity to a specific drug presumes the identification of genetic as well as epigenetic mechanismsThe therapeutic outcome (i. e. tumor eradication or growth retardation) is determined not as much by one factor but by their combinations. For a drug to be efficient, a coincidence of conditions must take place: cell death would be especially pronounced if inactivation of one gene is accompanied by the second mechanism (“synthetic lethality”). Increasing evidence points to the mutational “burden” of individual tumors, meaning the pairs of synthetic lethal genes (Du et al., 2023; Previtali et al., 2024). For tumors with a transcriptional “burden” (in particular, pediatric malignanices), genetic mutations are less important than epigenetic dysregulation (Comitani et al., 2023). Using Drosophila as a model, it was demonstrated that even temporary transcriptional disturbances can be carcinogenic without genetic alterations (Parreno et al., 2024).

Molecular correlations established in the present study determine cell fate upon inactivation of supertarget genes, thereby providing the mechanistic basis for rational drug combinations to treat “mutational” and “transcriptional” tumors.

## Conflict of interest

The authors declare no conflict of interest.
